# Clinical epidemiological characteristics of nitrous oxide abusers: A single‐center experience in a hospital in China

**DOI:** 10.1002/brb3.2416

**Published:** 2021-11-13

**Authors:** Yueyue Li, Jing Dong, Ran Xu, Fanfan Feng, Weihao Kan, Hongmei Ding, Xiaolong Wang, Yujie Chen, Xin Wang, Shiguang Zhu, Ruiguo Dong

**Affiliations:** ^1^ Clinical College Xuzhou Medical University Xuzhou China; ^2^ Department of Neurology The Affiliated Hospital of Xuzhou Medical University Xuzhou China

**Keywords:** epidemiology, neuropathy, nitrous oxide, public health, subacute combined degeneration, vitamin B12

## Abstract

**Purpose:**

This study investigated the clinical epidemiological characteristics of nitrous oxide (N_2_O) abusers in a hospital in China, which have not been systematically reported.

**Methods:**

The characteristics of patients abusing N_2_O who were examined and treated at the Affiliated Hospital of Xuzhou Medical University from January 2017 to December 2020 were analyzed.

**Results:**

A total of 61 patients (average age: 21.7 ± 3.2 years; 42 male and 19 female) were enrolled; 60.7% of the patients had an education level of high school or lower, and most (59.0%) had no stable occupation. The mean exposure time was 8.5 ± 7.7 months (range: 1–36 months). Only 52.5% of the abusers reported the physician of the relevant exposure history at the first time of visiting the doctor. The main clinical type was mixed (49.2%). The most common clinical manifestation was distal limb numbness (80.3%). The most frequent outcome was peripheral neuropathy (59%) and subacute combined degeneration (36%). Serum homocysteine level was elevated in 67.5% (27/40) of the patients, while 44.4% (20/45) showed reduced vitamin B12. Note that 61% (22/36) showed abnormal signals in the posterior or lateral funiculus of the spinal cord, and 97% (31/32) of the patients showed peripheral nerve damage by electromyography. In all cases, symptoms were alleviated after halting N_2_O intake and receiving nutritional neurotherapy.

**Conclusions:**

N_2_O abuse can lead to nervous system damage, especially peripheral nerve and spinal cord damage. A full understanding of its clinical epidemiological characteristics is helpful for clinicians to make a timely and clear diagnosis.

## Graphical abstract

Long‐term abuse of N_2_O mainly leads to nervous system damage by affecting the metabolism of vitamin B12, which is seriously harmful to the health of some teenagers. There are certain rules and characteristics in the clinical and epidemiological characteristics. A full understanding of these characteristics is helpful for clinicians to make timely and accurate diagnosis and treatment.

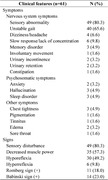

## 1 INTRODUCTION

Nitrous oxide (N_2_O) (i.e., laughing gas) is widely used in clinical practice for analgesia, sedation, and anesthesia. In recent years, an increasing number of young people have been abusing N_2_O in their pursuit of stimulation, with a concomitant increase in the incidence of related neurologic diseases and irreversible nerve damage. However, the risks associated with N_2_O abuse—which include subacute combined lesions of spinal cord, toxic encephalopathy, peripheral neuropathy, and mental disorders—are not fully recognized by society and clinicians. Previous studies have focused on the pathogenesis and clinical characteristics of diseases caused by N_2_O abuse, and there have been few epidemiologic investigations despite the importance of data from such studies for the development and implementation of effective interventions by health authorities (Bao et al., 2020; Garakani et al., 2016; Kaar et al., 2016; Lan et al., 2019; Li et al., 2016; Tuan et al., 2020). In order to collect epidemiologic data on N_2_O abuse, the present study analyzed the general epidemiological and clinical characteristics of 61 patients who were abusing N_2_O and were treated at the Affiliated Hospital of Xuzhou Medical University from January 2017 to December 2020. Our findings can improve the clinical diagnosis and treatment of patients who abuse N_2_O and guide health policies to address this problem.

## 2 METHODS

### 2.1 Patients

Between January 2017 and December 2020, 71 patients who were abusing N_2_O were treated at the Affiliated Hospital of Xuzhou Medical University. After excluding patients with incomplete baseline data and those who met the exclusion criteria, 61 patients were enrolled in the present study (20 outpatients and 41 inpatients).

### 2.2 Inclusion and exclusion criteria

At present, there is no unified diagnostic standard for N_2_O abuse. The inclusion criteria used to select patients for this study were as follows: (1) a clear history of N_2_O inhalation over the course of the disease; (2) clinical manifestations and signs of nervous system damage after N_2_O abuse that could not be attributed to other nervous system diseases; and (3) complete baseline data were available. The exclusion criteria were as follows: (1) a history of neurologic and mental illness before exposure to N_2_O and (2) use of other addictive drugs during the period of N_2_O abuse.

### 2.3 Data collection

Based on studies on methamphetamine‐related mental symptoms conducted in China (M. F. Su, Liu, et al., 2018), the following information on patients abusing N_2_O was collected: household registration, sex, age, education level, occupation, exposure time, first visit, final diagnosis, and classification. Additionally, the main clinical symptoms and signs were as follows; laboratory (including complete blood count test, vitamin B12, folate, homocysteine, glycohemoglobin, thyroid function, syphilis, HIV, viral antibodies, *MTHFR* gene, and cerebrospinal fluid), imaging, and neuroelectrophysiologic findings; and treatments and related data were summarized and analyzed.

### 2.4 Data processing

Quantitative data were presented as means and SD (x ± s). Qualitative data were described as counts or percentages, and intergroup comparisons were performed by the Fisher test. Non‐normally distributed data were expressed as medians and interquartile range (IQR). *p* < .05 was considered statistically significant. Statistical analysis was performed using SPSS 24.0 (SPSS Inc., Chicago, IL, United States).

## 3 RESULTS

### 3.1 Epidemiology of patients abusing N_2_O

**FIGURE 1:**
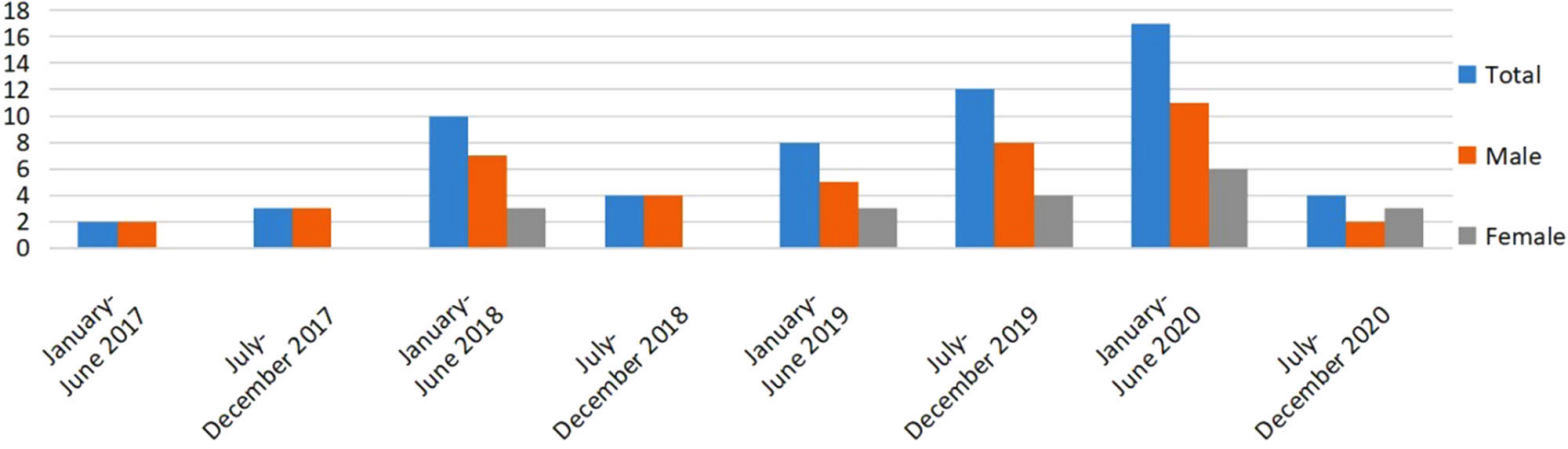
Number of patients abusing nitrous oxide (N_2_O) from January 2017 to December 2020

The number of patients abusing N_2_O treated at our hospital showed a generally increasing trend from January 2017 to June 2020, although it decreased thereafter (Figure 1).

### 3.2 Demographic and clinical characteristics of the study population

**FIGURE 2:**
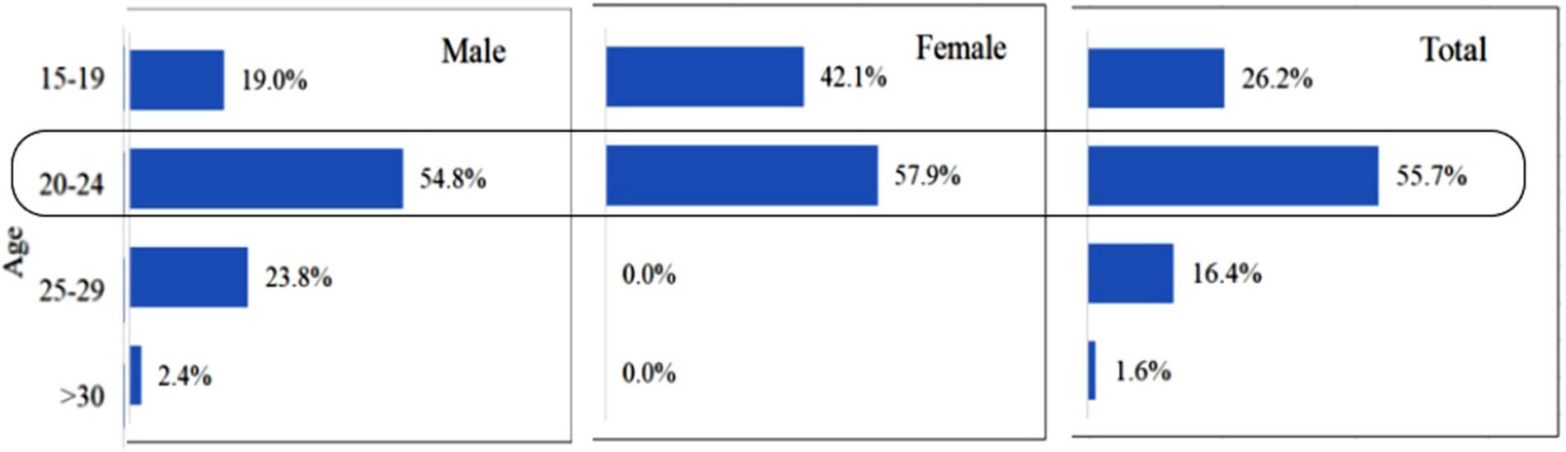
Age distribution of nitrous oxide (N_2_O) abusers according to sex

The demographic characteristics of the patients are shown in Table 1. Of the 61 patients who met the inclusion criteria, 42 (70%) were male and 19 (30%) were female. The age range was 15−31 years, with an average age of 21.7 ± 3.2 years (22.6 ± 3.1 years for males and 19.7 ± 2.6 years for females). N_2_O abuse was more common in males than in females in all age groups (Figure 2); the highest incidence of N_2_O abuse was in the 20−24 year age group in both sexes, and all of the female patients were <24 years old.

**TABLE 1:** Characteristics of the study population

Characteristic	Total (*n* = 61)	Male (*n* = 42)	Female (*n* = 19)
Age (years)			
15−19	16 (26.2)	8 (19.0)	8 (42.1)
20−24	34 (55.7)	23 (54.8)	11 (57.9)
25−29	10 (16.4)	10 (23.8)	0 (0.0)
≥30	1 (1.6)	1 (2.4)	0 (0.0)
Marital status			
Married	13 (21.3)	13 (31.0)	0 (0.0)
Unmarried	48 (78.7)	29 (69.0)	19 (100.0)
Residence			
Rural	24 (39.3)	18 (42.9)	6 (31.6)
Urban	37 (60.7)	24 (57.1)	13 (68.4)
Education level			
Bachelor's degree or higher	5 (8.2)	3 (7.1)	2 (10.5)
Junior college	19 (31.1)	14 (33.3)	5 (26.3)
High school	21 (34.4)	14 (33.3)	7 (36.8)
Middle school or lower	16 (26.2)	11 (26.2)	5 (26.3)
Occupation			
Student	9 (14.8)	6 (14.3)	3 (15.8)
Clerk	10 (16.4)	7 (16.7)	3 (15.8)
Self‐employed	20 (32.8)	13 (31.0)	7 (36.8)
Unemployed	16 (26.2)	11 (26.2)	5 (26.3)
Unknown	6 (9.8)	5 (12.0)	1 (5.3)

Note: Data represent *n* (%).

A minority of patients (5/61, 8.2%) had a bachelor's degree or higher education level. Most of the patients (36/61, 59.0%) were self‐employed or unemployed, and the rest had stable occupations. Most of the patients (78.7%) were unmarried.

There were 54 patients (88.5%) who had local household registration and seven (11.5%) with nonlocal registration; 37 (60.7%) cases were from an urban area, whereas 24 (39.3%) were from a rural area.

The duration of N_2_O exposure ranged from 1 to 36 months (mean: 8.5 ± 7.7 months), and 35 patients (57.4%) had abused N_2_O for >6 months. The duration of hospitalization among inpatients ranged from 3 to 19 days (8.7 ± 3.5 days). Except for three cases in the emergency department, the patients were treated in the neurology department for the first time.

### 3.3 Diagnosis

The initial diagnosis in 65.6% of the patients was peripheral neuropathy; only 32 (52.5%) informed the physician of their history of exposure at the first visit. The exposure history of nearly half of the patients was revealed only after admission, and four cases were misdiagnosed as Guillain–Barré syndrome and received hormone or gamma globulin treatment (Table 2). As there is no standard classification for patients who abuse N_2_O, we classified the patients based on a combination of criteria from previous reports and clinical data as follows (Table 3): (1) peripheral nerve type (involving only the peripheral nerve); (2) spinal cord type (involving only the spinal cord); (3) mixed type (involving both peripheral nerve and spinal cord); (4) psychiatric type (mental symptoms without peripheral or central nervous system [CNS] involvement); and (5) other type (involving autonomic nerves or could not be classified). The final diagnosis was peripheral neuropathy in 36 cases (59%), subacute combined degeneration of the spinal cord in 22 cases (36%), and toxic encephalopathy in three cases.

**TABLE 2:** Preliminary diagnosis for patients with different exposure histories

Preliminary diagnosis	N
Patient learned of exposure history upon visiting a doctor (*n* = 32)	
Peripheral neuropathy	23
Spinal cord degeneration	3
Toxic encephalopathy	3
Myelopathy	1
Muscle weakness to be investigated	1
Limb numbness to be investigated	1
Exposure history revealed after patient was admitted to the hospital (*n* = 29)	
Peripheral neuropathy	17
Myelopathy	5
Guillain–Barré syndrome	4
Muscle weakness to be investigated	3

**TABLE 3:** Clinical classification

Type	N (%)
Peripheral nerve	19 (31.1)
Spinal cord	0 (0.0)
Mixed	30 (49.2)
Psychiatric	3 (4.9)
Other	9 (14.8)

### 3.4 Main clinical manifestations

Neurologic lesions caused by N_2_O were observed in our cohort. The top symptoms were numbness (80.3%) and limb weakness (65.6%), which was a decrease in distal muscle strength in most instances (Table 4). Other neurologic manifestations included hypoesthesia, ataxia, pyramidal tract damage, deep reflex loss, somnolence, memory loss, and hallucinations. A small number of patients exhibited sore throat, tinnitus, palpitation, defecation disturbance, and other physiologic symptoms.

**TABLE 4:** Symptoms and signs of patients in this study

Clinical features (*n* = 61)	N (%)
Symptoms	
Nervous system symptoms	
Sensory abnormality	49 (80.3)
Unstable gait	40 (65.6)
Dizziness/headache	4 (6.6)
Slow response/lack of concentration	6 (9.8)
Memory disorder	3 (4.9)
Involuntary movement	1 (1.6)
Urinary incontinence	2 (3.2)
Urinary retention	2 (3.2)
Constipation	1 (1.6)
Psychosomatic symptoms	
Anxiety	2 (3.2)
Hallucination	3 (4.9)
Sleep disorder	3 (4.9)
Other symptoms	
Chest tightness	3 (4.9)
Pigmentation	1 (1.6)
Tinnitus	1 (1.6)
Edema	2 (3.2)
Sore throat	1 (1.6)
Signs	
Sensory disturbance	49 (80.3)
Decreased muscle power	35 (57.3)
Hyporeflexia	30 (49.2)
Hyperreflexia	6 (9.8)
Romberg sign (+)	11 (18.0)
Babinski sign (+)	14 (23.0)

### 3.5 Findings from supplementary examinations

#### 3.5.1 Laboratory examination

Of the 45 patients who underwent serum vitamin B12 analysis, 44.4% (20/45) showed reduced vitamin B12. Of the 40 patients who underwent serum homocysteine (HCY) analysis, 27 (67.5%) had elevated levels. Hematologic examination revealed anemia in three patients (Table 5). Mutation of the methylene tetrahydrofolate reductase (*MTHFR*) was detected in six cases (TT, *n* = 3; CC, *n* = 2; and CT, *n* = 1). Five patients were positive for syphilis antibody; the syphilis titers of four patients were 1:2, 1:16, 1:32, and 1:32, respectively, and one patient has a positive result for the *Treponema pallidum* particle agglutination assay. Cerebrospinal fluid examination was performed in 11 patients, and elevated protein levels were detected in four, but there were no specific changes.

**TABLE 5:** Laboratory data for this study

Laboratory date	Mean ± SEM/N (%)	Range
Hemoglobin (normal 110–150 g/dl)	135.5 ± 12.3	100–158
Anemia	3 (4.9)	
Vitamin B12 (normal 187–883 pg/L)	233.50 (143.75, 477.75)	94–725
Decreased	20 (44.4)	
HCY (normal 5.45–16.20 μmol/L)	23.53 (14.67, 47.33)	8.54–50
Increased	27 (67.5)	

Abbreviation: HCY, homocysteine.

#### 3.5.2 Imaging examination

**FIGURE 3:**
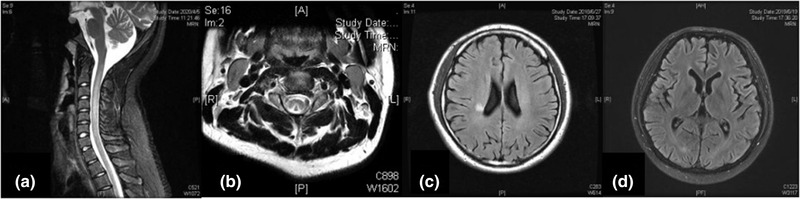
Representative magnetic resonance (MR) images of central nervous system abnormalities in patients abusing nitrous oxide (N_2_O). (a) MR image of cervical vertebrae showing a high T2 signal. (b) T2‐weighted image from cervical MRI showing an inverted “V” sign in the posterior funiculus of the spinal cord. (c) magnetic resonance imaging (MRI) of the skull showing a T2FLAIR hyperintense signal in the right radiation coronal area. (d) Brain MRI showing brain atrophy

A total of 36 patients underwent magnetic resonance imaging (MRI) examination of the skull and cervicothoracic vertebrae. Among the 36 patients, abnormal spinal cord signal was found in 22 cases (61%) and typical spinal cord degeneration in 13 cases (36%), which showed long T2 signal at the proximal end of the spinal cord and inverted “V” shape on T2‐weighted images. In 20 cases (91%), there was an involvement of ≥3 spinal cord segments. Cranial MRI showed lacunar foci in four cases and cerebral atrophy in one case (Figure 3).

In 22 cases of abnormal spinal cord MRI, 16 cases (72.7%) had exposure time ≤6 months, six cases > 6 months; 14 cases had normal spinal cord MRI, 11 cases (78.57%) had exposure time > 6 months, three cases (27.27%) ≤6 months. There was a significant difference between the two groups (*p* < .05) (Table 6).

**TABLE 6:** Relationship between abnormal signal of spinal cord and exposure time

Exposure time	Abnormal	Normal	Total	Abnormal rate
≤6 m	16	3	19	84.2%
>6 m	6	11	17	35.3%
*p*‐Value				<.05

#### 3.5.3 Electromyography examination

Electromyography (EMG) was performed in 32 patients; 31 (97%) showed peripheral nerve damage, and most had motor and sensory involvement (Table 7).

**TABLE 7:** Neuroelectrophysiologic manifestations in nitrous oxide (N_2_O) abuse patients

Parameter	n/N (%)
Motor conduction	
Abnormal number	28/32 (88%)
CMAP amplitude decrease	25/32 (78%)
MCV decrease	27/32 (84%)
Prolonged exercise latency	14/32 (44%)
Sensory conduction	
Abnormal number	31/32 (97%)
SNAP amplitude decrease	17/32 (53%)
SCV decrease	31/32 (97%)
F wave	
Abnormal number	31/32 (97%)
Decrease or disappearance	24/32 (75%)
Prolonged latency period	27/32 (84%)
H reflex	
Abnormal number	24/24 (100%)
Decrease or disappearance	6/24 (25%)
Prolonged latency period	18/24 (75%)
Denervation potential detected by EMG	21/32 (67%)

Abbreviations: CMAP, compound muscle action potential; EMG, electromyogram; MCV, motor conduction velocity; SNAP, sensory nerve action potential; SVC, sensory conduction velocity.

### 3.6 Treatment and outcome

To halt N_2_O intake, all patients received nutritional neurotherapy such as vitamin B12 or mecobalamin injection. Three patients with syphilis titers of 1:16, 1:32, and 1:32 were treated with penicillin after dermatology consultation. The 61 patients were followed up for 1−3 months after treatment; two patients had dyskinesia (mainly decreased distal muscle strength), one had residual anxiety, and the others had no obvious sequelae. No relapse occurred in our cohort during the follow‐up.

## 4 DISCUSSION

Since diseases caused by N_2_O abuse were first reported in China in 2016, the number of cases has been increasing yearly. Long‐term abuse of N_2_O not only causes craniocerebral lesions, subacute combined degeneration (SCD), and peripheral neuropathy but also rare symptoms such as skin pigmentation, chest tightness, and auditory hallucinations. According to the 2014 Global Drug Survey report, N_2_O abuse is increasing globally (Kaar et al., 2016). In Asian countries, N_2_O abuse is becoming more prevalent, especially among teenagers (Lan et al., 2019). Whipped cream canisters designed for the food industry, which are also called “whippets” or “laughing gas bombs,” are the main source of N_2_O for recreational use (Butzkueven & King, 2000). However, to date, there have been no systematic investigations on this topic in China. As many doctors have limited understanding of the epidemiology of N_2_O abuse and the clinical characteristics of associated diseases, it may be underdiagnosed or misdiagnosed. Additionally, relevant laws and regulations are inadequate. At present, the relevant domestic laws and regulations do not include nitrous oxide in the ranks of drugs, only as dangerous chemicals for control. Teenagers who abuse nitrous oxide may be subject to administrative penalties for public security. However, due to the lag of management, some lawbreakers take advantage of loopholes to carry out illegal trafficking. Therefore, more up‐to‐date and comprehensive epidemiologic and clinical data on N_2_O abuse in China are needed to guide policies as well as prevention and control efforts.

Our data showed that the number of patients seeking medical assistance for symptoms related to N_2_O abuse has been increasing yearly at our hospital; however, the number of patients decreased markedly after June 2020. There are a few possible reasons for this decline. First, during the coronavirus disease of 2019 (COVID‐19) pandemic in China, parties and large‐scale activities such as festivals—venues that could encourage recreational N_2_O use (van Amsterdam et al., 2015)—were prohibited, which may have temporarily reduced the rate of N_2_O abuse. Second, because adverse reactions may not occur in the early stages of abuse, most of the patients do not seek treatment. Third, it could also reflect the impact of public education and increased awareness of N_2_O abuse as well as the efforts by law enforcement agencies to thwart the illegal sale of N_2_O.

In our study population, 89.3% of the patients were registered locally and only a small number came from provinces and cities adjacent to Xuzhou. We also found that there was no significant difference in the number of patients from urban and rural areas. However, given the relatively small sample size in this study, it is possible that there is a difference in the rate of N_2_O abuse according to the type of community (i.e., urban vs. rural).

The data show that the patients with N_2_O abuse have the following demographic characteristics: (1) the overall age is younger, there are more men than women, and the average age of women is lower than that of men; (2) the proportion of unmarried patients is higher; (3) the overall level of education is not high; and (4) most of the patients have no stable occupation. In previous studies, the patients of N_2_O abuse were mainly adolescents (Bao et al., 2020; Garakani et al., 2016; Li et al., 2016; Tuan et al., 2020), and no significant gender differences were mentioned. However, whether there are significant gender differences in patients with N_2_O abuse still needs a large sample study. Women are more likely to use psychotropic drugs earlier (He et al., 2013), which explains the average age difference between men and women. The fact that there are more unmarried women than men is considered to be related to the fact that the average age of men is higher than that of women. Based on these data, young people who are single, have a low level of education, and lack stable employment are the target demographic for education regarding the risks of N_2_O abuse. Five of our patients were positive for syphilis by virologic examination. Previous studies have reported high rates of AIDS and syphilis among drug users as they are more likely to have unprotected sex (S. Su, Mao et al., 2018). This is the first report of the positive rate of syphilis among N_2_O abusers. However, it still needs a lot of data to study whether N_2_O abusers have the same high infection rate of syphilis as other drug abusers.

In cases of N_2_O abuse, a clear history of exposure is particularly important for early diagnosis and treatment. We found that most of the patients (95.1%) were visiting the neurology department for the first time but 47.5% of the abusers did not report the physician of the relevant exposure history at the first time of visiting the doctor. Most of these cases were initially diagnosed as peripheral neuropathy, five were admitted to the hospital with myelopathy, and four were misdiagnosed as Guillain–Barré syndrome and underwent related examination and treatment. In many cases, the history of exposure was unknown even after many outpatient visits, possibly because of the refusal of some patients to provide this information and a lack of awareness of symptoms on the part of clinicians.

According to our N_2_O abuse‐related disorder classification, peripheral nerve with spinal cord injury is the most common type of injury. Brain parenchyma lesions have been previously reported, but there were no cases with spinal cord or brain parenchyma involvement in our study (Assaf et al., 2020; Bajaj et al., 2018). Because patients’ self‐reports of exposure may not be reliable, we were unable to compare the clinical data of different types of patients; therefore, it is unclear whether the peripheral nerve damage caused by N_2_O precedes CNS damage, or whether patients with CNS involvement have worse prognosis. Additionally, the psychiatric manifestations of N_2_O abuse including delusions, hallucinations, and mental confusion should not be ignored (Assaf et al., 2020; Bajaj et al., 2018). Some studies have also reported more severe mental symptoms leading to suicide or violence; professional psychiatric evaluation is recommended in such cases (Chien et al., 2020).

In our study, 91.8% of the patients were ultimately diagnosed with peripheral neuropathy or subacute combined SCD, which is in line with other reports (Garakani et al., 2016; Li et al., 2016). The main neurologic symptoms in our patients were limb weakness, numbness, and sensory abnormalities, while a subset exhibited mental abnormalities and autonomic nervous dysfunction (bladder and sexual dysfunction). In general, short‐term exposure to N_2_O is not associated with neurologic complications unless the patient has vitamin B12 deficiency or an absorption disorder. However, long‐term intake of N_2_O >80 g/day can increase the risk of diseases (Alt et al., 2011; Cheng et al., 2013).

It is thought that long‐term abuse of N_2_O causes nervous system dysfunction by altering vitamin B12 metabolism (Lassen et al., 1956). Vitamin B12 mainly exists in two active forms: adenosine cobalamin and methylcobalamin. The latter is an important cofactor of methionine synthase (MS), the rate‐limiting enzyme in the conversion of HCY to methionine (Met). N_2_O irreversibly oxidizes cobalt ion (Co+) in vitamin B12, thereby inactivating and inducing the excretion of cobalamin from the body, which results in the inhibition of MS activity, accumulation of HCY, and decreased Met synthesis (Flippo & Holder, 1993; Healton et al., 1991; Reynolds, 2006). Under normal circumstances, Met is converted into S‐adenosylmethionine (SAM); impairment of SAM and Met synthesis can lead to the failure of myelin methylation and demyelinating changes in the spinal cord (Healton et al., 1991). Additionally, HCY accumulation was shown to exert toxic effects by enhancing oxidative stress and activation of N‐methyl‐d‐aspartate receptor in neurons, leading to neuronal death. N_2_O is itself neurotoxic and can affect cortical function. We found that blood HCY was elevated in 67.5% of the patients, and vitamin B12 was decreased in 44.4%, consistent with previous work that elevated blood HCY levels were more diagnostic than vitamin B12 deficiency (Ahn & Brown, 2005). Interestingly, 31.1% of the patients had an elevated serum vitamin B12 level when they were admitted to our hospital; this may be because they had already received vitamin B12 treatment or were aware that taking vitamin B12 could prevent complications related to N_2_O inhalation. The cerebrospinal fluid examination in our patients showed no obvious changes, but it is nonetheless important for the differential diagnosis of N_2_O‐related disease.

Human *MTHFR* gene is located on chromosome 1p36.3 and contains 11 exons, which are responsible for encoding important enzymes involved in folic acid/HCY metabolism. Its transcriptional product is a 77 kDa protein that can catalyze the demethylation of 5,10‐methyltetrahydrofolic acid to 5‐methyltetrahydrofolic acid, which can be reduced to 5‐methyltetrahydrofolic acid with biological functions. 5‐Methyltetrahydrofolic acid as a methyl donor induces HCY re‐methylation to methionine. The amino acid mutation of *MTHFR* gene may affect the structure and function of the gene coding products. So far, many types of *MTHFR* gene mutations have been reported. Among them, C667T mutation has a relatively high frequency all over the world. A mutation in codon 667 (CC→CT or TT) decreases MTHFR activity (Goyette et al., 1994), dysregulates folate metabolism, increases plasma HCY level (Liew & Gupta, 2015), and is an independent predictor of coronary heart disease and stroke. It was reported that plasma HCY was higher after N_2_O anesthesia in patients with *MTHFR* gene mutation than in those with the wild‐type gene (Nagele et al., 2008); and neonates with MTHFR deficiency developed severe neurologic symptoms and died after inhaling a small amount of N_2_O (Erbe & Salis, 2003). We speculate that patients with a mutated (CT or TT) *MTHFR* gene are more susceptible to the effects of N_2_O or have a worse clinical outcome than those with the wild‐type (CC) gene. In this study, *MTHFR* gene was detected in six patients. However, in view of the differences in nitrous oxide exposure time and intake, and the relatively small number of cases included in this study, it remains to be determined whether *MTHFR* gene polymorphism is related to the clinical severity and prognosis of N_2_O abuse cases.

SCD is a neurologic complication of vitamin B12 deficiency. Spinal cord MRI can reveal long or spotted abnormal signals in the posterior or lateral funiculus that are generally associated with the cervical and upper thoracic spinal cord and occasionally the lateral cord, but are rarely associated with the anterior funiculus, which may reflect how demyelination progresses in the dorsal spinal cord (Jain et al., 2014). In our study, 53% of the patients showed abnormal signals in the cervical spinal cord, mostly associated with only the posterior funiculus, which is in agreement with previous findings about SCD (Cao et al., 2018). A previous study had shown that the evolution of conventional MRI findings lagged compared to the clinical manifestation because T2‐weighted imaging is not sensitive enough to show cytotoxic edema, suggesting clinical‐radiological dissociation (Jiang & Shang, 2020); moreover, we also found that the incidence of MRI lesions is higher in patients with an N_2_O exposure time of ≤6 months. Thus, while spinal cord MRI can be used as an auxiliary tool along with medical history, clinical features, and hematologic examination in the diagnosis and treatment of the patients who abuse N_2_O, it may not be suitable for evaluating N_2_O‐induced SCD and cannot predict clinical outcomes. Some of our patients also had abnormal signals in the cranial MRI including lacunar foci in four patients and brain atrophy in one patient, which may indicate white matter degeneration.

Peripheral nerve damage is common in N_2_O abusers. Demyelination with axonal damage has been described in previous studies (Bao et al., 2020; Li et al., 2016). Neuroelectrophysiologic examination revealed multiple peripheral neuropathies in 97% of the patients related to movement (88%) and sensation (97%), as well as abnormal F wave and H reflex. Thus, N_2_O abuse causes peripheral nerve damage in most of the cases.

There are no guidelines for the treatment of nervous system damage caused by N_2_O abuse; the main recommendations are to halt N_2_O intake and use vitamin B12 supplementation (Keddie et al., 2018). Met can also be used as an adjuvant (Stacy et al., 1992). Methylprednisolone combined with vitamins was shown to alleviate SCD caused by N_2_O abuse (Zhang et al., 2021). Most of the patients have a good prognosis after timely treatment, and only a small percentage of patients continue to experience mild sensory or motor disorder. However, chronic N_2_O exposure can lead to irreversible nerve damage or even sudden death (Layzer et al., 1978). Some studies have demonstrated that vitamin B12 did not prevent demyelination in patients who consistently abused N_2_O (Blair et al., 2019). During the follow‐up, clinical symptoms were relieved to varying degrees in 96.7% of our patients; additionally, there were no instances of relapse, suggesting that none of the patients were addicted to N_2_O.

This study had some limitations. First, as it was a retrospective study, some of the patients’ basic data as well as their prognosis were learned by telephone follow‐up, which may have introduced inaccuracies. Secondly, the specific amount of N_2_O consumed by patients could not be calculated. Thirdly, not all patients underwent spinal MRI and EMG, which could have provided more detailed information on functional consequences of N_2_O abuse.

To sum up, patients with N_2_O abuse have certain clinical epidemiological characteristics. Long‐term abuse of N_2_O mainly leads to nervous system injury by affecting the metabolism of vitamin B12, and peripheral nerve and spinal cord injury is most common. Clinicians must understand the characteristics of neurological diseases related to N_2_O abuse, take the initiative to inquire about the history of exposure to suspicious patients, and improve the relevant examination in time.

## CONFLICT OF INTEREST

The authors declare no conflict of interest.

## AUTHOR CONTRIBUTIONS

Yueyue Li, Ruiguo Dong, Jing Dong, and Ran Xu conceived and designed the study. Yueyue Li, Jing Dong, Hongmei Ding, and Xin Wang acquired the data. Yueyue Li, Jing Dong, Ran Xu, Fanfan Feng, and Weihao Kan analyzed and interpreted the data. All authors contributed to the writing of the manuscript, read, and approved the final version.

### PEER REVIEW

The peer review history for this article is available at https://publons.com/publon/10.1002/brb3.2416

## Data Availability

The data that support the findings of this study are available from the corresponding author upon reasonable request.
